# MLL3 suppresses tumorigenesis through regulating TNS3 enhancer activity

**DOI:** 10.1038/s41419-021-03647-2

**Published:** 2021-04-06

**Authors:** Jun-Yi Zheng, Chen-Yu Wang, Chuan Gao, Qiong Xiao, Cheng-Wei Huang, Min Wu, Lian-Yun Li

**Affiliations:** 1 Frontier Science Center for Immunology and Metabolism, Wuhan University, Wuhan, Hubei 430072 China; 2 College of Life Sciences, Hubei Key Laboratory of Cell Homeostasis, Hubei Key Laboratory of Developmentally Originated Disease, Hubei KeyLaboratory of Enteropathy, Wuhan University, Wuhan, Hubei 430072 China

**Keywords:** Tumour-suppressor proteins, Chromosomes

## Abstract

MLL3 is a histone H3K4 methyltransferase that is frequently mutated in cancer, but the underlying molecular mechanisms remain elusive. Here, we found that *MLL3* depletion by CRISPR/sgRNA significantly enhanced cell migration, but did not elevate the proliferation rate of cancer cells. Through RNA-Seq and ChIP-Seq approaches, we identified *TNS3* as the potential target gene for MLL3. *MLL3* depletion caused downregulation of H3K4me1 and H3K27ac on an enhancer ~ 7 kb ahead of TNS3. 3C assay indicated the identified enhancer interacts with *TNS3* promoter and repression of enhancer activity by dCas9-KRAB system impaired *TNS3* expression. Exogenous expression of *TNS3* in *MLL3* deficient cells completely blocked the enhanced cell migration phenotype. Taken together, our study revealed a novel mechanism for MLL3 in suppressing cancer, which may provide novel targets for diagnosis or drug development.

## Introduction

Enhancers are cis-acting elements for transcription factor binding and activate transcription over a long distance. One of the important features for enhancers is that specific patterns of histone modifications occupy the surrounding nucleosomes, which are brought by pioneer factors^1^. Histone modifications are critical for enhancer activity and now often used as hallmarks to identify enhancers^2–5^. Usually, H3K4me1 is the basic mark to label enhancers. If H3K27ac is also present, the enhancer is active; if H3K27me3 occupies the region instead of H3K27ac, the enhancer is poised^6–9^. Nowadays, ChIP-Seq of histone modifications is often used to annotate enhancers. For example, Creyghton et al. predicted 25,036 enhancers in mESC by identifying chromatin regions with enriched H3K4me1 but not H3K4me3. They found that the proximal enhancer genes lacking H3K27ac showed lower expression than the average proximal genes, indicating H3K27ac as an active enhancer hallmark^10^. H3K27ac enriched regions in the intergenic chromatin are now used to identify active enhancers^3,11,12^. Generally, H3K4me3 marks the gene transcription start sites (TSS)^13^. However, it was reported recently that H3K4me3 also exists in some overactive enhancers, and may be associated with tumorigenesis^14,15^.

Genetic and epigenetic alterations usually happen together in cancer cells. It has been reported that epigenetic genes are one of the largest groups of frequent mutated genes in cancer^16,17^. Interestingly, many histone modification enzymes involved in enhancer regulation have high mutation rates. TCGA data indicate that in multiple types of cancer MLL3/4 (lysine methyltransferase 2C/D, or mixed-lineage leukemia 3/4) complex and p300/CBP (E1A binding protein p300/ CREB binding protein) histone acetyltransferase are frequently mutated, which are key enzymes for H3K4me1 and H3K27ac, respectively^18^. Mutation of MLL3 results in the defect of BRCA1 associated protein 1 (BAP1) recruitment to the enhancers of tumor-suppressor genes, which represses enhancer activity and promotes tumorigenesis^19^. In breast cancer, the defect of lysine demethylase 5C (KDM5C) causes H3K4 trimethylation and overactivation of oncogene enhancers, which then promotes oncogene expression and tumorigenesis^15^. Moreover, other histone modifications or associated proteins regulate the enhancer activity of specific signaling pathways, which then affect the expression of target genes and tumorigenesis^20,21^. For example, histone demethylase KDM3A removes H3K9me2 on enhancers and promotes the gene expression downstream of the hippo pathway^21^. Recently, the concept of super enhancers was raised and it has been hypothesized that super enhancers on oncogenes are associated with tumorigenesis^11,22,23^. Therefore, it is critical to fully understand the roles and mechanisms of enhancer regulation in tumorigenesis and metastasis.

MLL3 is a methyltransferase for H3K4 and forms a complex with multiple subunits, including WD repeat domain 5 (WDR5), RB binding protein 5 (RBBP5), ASH2 like (ASH2L), dpy-30 histone methyltransferase complex regulatory subunit (DPY30), PAX interacting protein 1 (PAXIP1), PAXIP1 associated glutamate-rich protein 1 (PAGR1) and lysine demethylase 6A (KDM6A)^24,25^. MLL3/4 complexes only contain weak activity to tri-methylation on H3K4^24^, but they are critical for H3K4me1 on enhancer loci^26–30^. MLL3 deficiency causes downregulation of H3K4me1 and H3K27ac on enhancers and impairs chromatin interaction between enhancer and promoter regions^28–31^. Interestingly, *MLL3* knockout only affects the expression of a small group of genes^32,33^. The relationship of H3K4me1, chromatin structure, and transcription still require more studies. Moreover, genome-wide association studies have revealed that *MLL3* is one of the most frequent mutated genes in many cancer types, but the mechanisms for MLL3 regulating tumorigenesis remain elusive. The current study identified *tensin 3* (*TNS3)* as a target gene for MLL3, which is critical for regulating cancer cell migration. Our work reveals important mechanisms for MLL3 in repressing tumorigenesis and metastasis.

## Materials and methods

### Reagents and cell lines

Antibodies recognizing β-Actin (Abclonal, AC026, RRID: AB_2768234), H3 (abcam, ab1791, RRID: AB_302613), H3K27ac (abcam, ab4729, RRID: AB_2118291), H3K4me1 (Cell Signaling Technology, #5326, RRID: AB_10695148), p53 (Santa Cruz, sc-126, RRID: AB_628082), p21 (Cell Signaling Technology, #2947, RRID: AB_823586), MDM2 (abcam, ab3110, RRID: AB_303518), PUMA (Cell Signaling Technology, #4976, RRID: AB_2064551), MLL3(Merck ABE1851) and TNS3 (Abclonal A7991, RRID: AB_2772674) were purchased from indicated vendors. PCR primers were custom synthesized by TSINGKE and siRNAs by GenePharma. Nutlin-3A (S8059) was purchased from Selleck.

MDA-MB-231 and HeLa cells were cultured in Dulbecco’s Modified Eagle Medium (Gibco) at 37 °C with 5% CO_2_, and U2OS and 769-P cells in RPMI 1640 (Gibco). Both mediums were supplemented with 10% FBS (Biological Industries) and 1% penicillin-streptomycin solution (HyClone). All cell lines were purchased from the Cell Bank of the Chinese Academy of Sciences.

### Immunoblot analysis

Cell lysates were prepared with SDS lysis buffer (50 Mm Tris-HCl pH 6.8, 4% SDS). Tissue lysates were prepared with RIPA buffer (50 Mm Tris-HCl pH 7.4, 150 Mm NaCl, 1% Triton X-100, 1% sodium deoxycholate, 0.5% SDS) and sufficiently grounded with homogenizer. Protein extracts were separated by SDS-PAGE and transferred to nitrocellulose filter membranes. After blocking with 5% milk in Tris-buffered saline and 0.1% Tween (TBS-T), the membranes were incubated with the desired primary antibodies overnight at 4 °C. Then the membranes were washed and incubation with the appropriate secondary antibodies at room temperature for 1 h. The blots were detected by Clarity Western ECL Substrate (BIO-RAD). For cell lysates, triplicate biological experiments were performed, and for animal tissues, at least three individual mice samples per group were used.

### Reverse transcription and quantitative PCR

For cell RNA extraction, cells were scraped down and collected with centrifugation. For tissue RNA extraction, 20 mg tissues were homogenized and collected with centrifugation. Total RNA was extracted with RNA extraction kit (Aidlab or CWBIO) according to the manufacturer’s manual. Approximately 1 μg of total RNA was used for reverse transcription with a first-strand cDNA synthesis kit (Vazyme). The resulted cDNA was then assayed with quantitative PCR (BIO-RAD CFX384TM). β-actin was used for normalization. The sequences of primers are provided in Sup. Table 3. Assays were repeated at least three times. Data were shown as average values ± SD or SEM. *P*-value was calculated using the student’s *t* test.

### Generation of knockout/knockdown cell line with CRISPR/Cas9 system

The small guide RNA (sgRNA) sequences were designed by using the CRISPR Design Tool (http://tools.genome-engineering.org), provided by Feng Zhang lab. The target sequences of human *MLL3* sgRNAs as follow: sgRNA1 5′- GCAGTTTTCCCCCTACTTCG-3′, sgRNA2 5′- ACTTGTGGTCAGCACTATCA-3′. GFP target sgRNA was used as the control, sgCtrl 5′- ACGGAGGCTAAGCGTCGCAA-3′. The oligo pairs containing the 20-nt guide sequences were annealed and ligated into the CRISPR plasmid (pLenti-v2-Cas9-gRNA). To construct knockdown cell lines, the plasmids of pLenti-v2-gRNA (Cas9 containing), psPAX2, and pMD2G were transfected into HEK-293T cells to produce the lentiviral particles. Then the supernatant was used to infect the desired cells, which were selected by puromycin and validated by western blotting.

### Repression of enhancer activity with dCas9-KRAB/sgRNA system

The sgRNA sequences were designed using the CRISPR Design Tool (http://tools.genome-engineering.org). The target sequences of human *TNS3* enhancer sgRNAs as follow: sgRNA1 5′- CCACTGTAATCTAAAGAGAG-3′, sgRNA2 5′- GAGAGAAGGATTGAGAAAGG-3′. The oligo pairs containing the 20-nt guide sequences were annealed and ligated into the pLH plasmid. The plasmids of pLH-gRNA, dCas9-KRAB, psPAX2, and pMD2G were transfected into HEK-293T cells to produce lentiviral particles. The supernatant was used to infect the desired cells, which were then selected with puromycin and hygromycin.

### ChIP assay

ChIP assay was performed as previously described^34,35^. In Brief, ~8 × 10^6^ U2OS cells were crosslinked with 1% formaldehyde for 10 min and quenched by glycine. The cells were washed three times with PBS and then harvested in ChIP digestion buffer (50 mM Tris-HCl, pH 7.6, 1 mM CaCl2, 0.2% Triton X-100) or ChIP lysis buffer (50 mM Tris-HCl, pH 8.0, 0.5% SDS, 5 mM EDTA). DNA was digested by MNase (Sigma) or sonicated to 150–300 bp. Supernatant was collected after extensive centrifugation. Digested DNA was diluted to five times of volume with ChIP digestion dilution buffer (20 mM Tris-HCl, pH 8.0, 150 mM NaCl, 2 mM EDTA, 1% Triton X-100, 0.1% SDS) and incubated with protein G beads and antibodies of H3K27ac, H3K4me1 or IgG at 4 °C overnight. Sonicated DNA was diluted to five times of volume with ChIP sonication dilution buffer (20 mM Tris-HCl, pH 8.0, 150 mM NaCl, 2 mM EDTA, 1% Triton X-100) and incubated with protein G beads and p53 antibody of or IgG at 4 °C overnight. The beads were washed five times with ChIP wash buffers and DNA was eluted by ChIP elution buffer (0.1 M NaHCO3, 1% SDS, 20 μg/ml proteinase K). The elution was incubated at 65 °C overnight for de-crosslinking and DNA was then extracted with DNA purification kit (TIANGEN DP214-03). The purified DNA was assayed by qRT-PCR (Biorad MyIQ) or high-throughput sequencing by Illumina Hiseq X Ten platform. The relative enrichment was normalized to input. ChIP-qPCR assays were repeated at least three times. Data were shown as average values ± SD and p-value were calculated using the student’s *t* test. The sequences of primers are in Sup. Table 3.

### Chromosome conformation capture (3C) assay

Approximately 1 × 10^6^ cells were crosslinked with 1% formaldehyde for 10 min and quenched by glycine. The cells were washed with PBS and lysed in cell lysis buffer (10 mM Tris-HCl, pH 7.5, 10 mM NaCl, 5 mM MgCl2, 0.1 mM EDTA, 1× complete protease inhibitor) at 4 °C for 30 min. Nuclei were collected after centrifugation at 400 × *g* at 4 °C for 5 min and removing the supernatant. The collected nuclei were digested with 400 U DpnII restriction enzyme (NEB) at 37 °C overnight. The digested nuclei were then added with 100 U of T4 DNA ligase (NEB) and incubated for 4 h at 16 °C followed by 30 min at room temperature. The samples were then added with 300 μg of proteinase K, incubated at 65 °C overnight for de-crosslinking, and purificated with DNA purification kit (TIANGEN DP214-03). The relative crosslinking frequencies between the *TNS3* enhancer and promoter were determined by qRT-PCR. One primer in the enhancer (E1) and five primers in the promoter region (P1–5) were designed. The relative cross-linking frequencies are calculated by normalizing to a primer pair (3C-N F, 3C-N R) without crossing the DpnII cut sites. Assays were repeated at least three times. Data were shown as average values ± SD and *p*-value were calculated using the student’s *t* test. The sequences of primers are in Sup. Table 3.

### Cell viability assay

Cell proliferation was analyzed by MTT assay. Cells were seeded on a 96-well plate about 2000 cells per well, incubated for 24, 48, or 72 h at 37 °C, respectively. Then 5 μl MTT (5 μg/μl) was added into each well, and incubated for 4 h at 37 °C. Four hundred microliters of lysate buffer (50% DMF + 30% SDS, pH 4.7) was added into each well followed by 4 h incubation at 37 °C. The absorbance at 570 nm was measured by Microplate System. The relative numbers of migrated cells were calculated by comparison to the absorbance of wells without adding cells. Assays were repeated at least three times. Data were shown as mean ± SD and *p*-value was calculated by the student’s *t* test.

### Cell migration and invasion assays

To examine cell migration, cells (U2OS 4 × 10^4^, 769-P 3 × 10^4^, HeLa and MDA-MB-231 8 × 10^4^ cells per well) were plated in the upper compartment of a 24-well Transwell tray (pore size 8 μm, BD Biosciences) in full medium lacking FBS. Medium supplemented with 10% FBS was used as a chemoattractant in the lower chamber. Cells were allowed to migrate through the intervening nitrocellulose membrane (8 μm pore size) during 12 h of incubation at 37 °C. For cell invasion examination, 4 × 10^4^ U2OS cells per well were plated in the upper compartment of a 24-well Transwell tray pre-coated with Matrigel (pore size 8 μm, Corning, 354480) instead, and cells were incubated at 37 °C for 18 h. Cells on the upper surface of the insert were then removed by a cotton swab. Cells migrated to the lower surface of the insert were fixed with methanol, stained with crystal violet, and then photographed. The numbers of migrated cells in five random fields under the microscope were counted for the quantitative analysis. Assays were repeated at least three times. Data were shown as mean ± SD and *p*-value was calculated by Student’s *t*-test.

### Wound healing assay

Cells were transfected with siRNAs for 48 h. Then 8 × 10^5^ cells were plated on 6-well plates for 24 h and wounded by scratching with a pipette tip. Subsequently, the cells were incubated with OPTI MEM medium without FBS at 37 °C for up to 36 h. Three random fields under the microscope were photographed for calculating the percentage of wound closure. Assays were repeated at least three times. Data were shown as mean ± SD and *p*-value was calculated by Student’s *t*-test.

### Xenograft experiments

Five-week-old female BALB/c nude mice were purchased from Beijing Vital River Laboratory Animal Technology Co. Ltd. The mice were grouped randomly and 8 × 10^6^ HeLa cells in 200 μl of PBS were injected in the flank regions. Ten points of five mice were injected for each cell line. Tumor volume was measured from 10 days post-inoculation and recorded every 4 days. All mice were sacrificed 32 days after cell injection, and tumors were harvested and weighed. All animal xenograft experiments were performed following the university laboratory animal guidelines and were approved by the Animal Experimentations Ethics Committee of Wuhan University.

### RNA-seq and data analysis

RNA-seq library was constructed by using Illumina TruSeq library construction kit (Vazyme, ND606). 4 µg total RNA was used for each sample, and libraries were sequenced using HiSeq X Ten for 150 bp paired-end sequencing. Quality control of mRNA-seq data was performed using Fatsqc and low-quality bases were trimmed. All RNA-seq data were mapped to the human reference genome hg19 by TopHat (version 2.1.1) and allow maximum 2 mismatch. The gene expression level was calculated by Cufflinks with default parameters. Genes with FPKM <1 in all samples were excluded. DEGs were identified by 1.5-fold change. Gene ontology analysis was performed using DAVID (https://david.ncifcrf.gov).

### Pipeline of ChIP-Seq data analysis

For ChIP-Seq analysis, Fastqc was used for raw data quality control. Cutadapt was used to remove law quality bases and library adaptor contamination (cutadapt -a AGATCGGAAGAGCACACGTCTGAACTCCAGTCAC -A AGATCGGAAGAGCGTCGTGTAGGGAAAGAGTGT -m 20). After quality control and data filtering, data were mapped to human reference genome hg19 using Bowtie2. Samtools was used to sort BAM file and filter duplicate reads. Only unique mapped reads were accepted for further analysis. MACS2 was used for ChIP-Seq peaks calling with *p* value cut-off 1e−10. Then HOMER annotatePeaks.pl was used to annotate ChIP-seq peaks compare to reference genome hg19.

For enhancer analysis, ChIP-seq data of siMLL3.1 and siMLL3.2 were merged together as siMLL3. Active enhancers were identified by regions with significant distal H3K27ac and H3K4me1 peaks (peak boundary 1.5 kb away from gene TSS). The enhancers were ranked by total signal of H3K4me1. The total signals of H3K4me1 and H3K27ac over the enhancer regions were then compared between siNC and siMLL3. The up or downregulated enhancers were defined by 1.2-fold change of total H3K4me1 and H3K27ac signals over the enhancer regions. Genes potential targeted by enhancers were identified by distance of the nearest enhancer to gene TSS within 10 kb.

### Bioinformatic analysis of clinical data

The clinical data analysis was performed by using the cBioportal (http://www.cbioportal.org/index.do), GEPIA (http://gepia.cancer-pku.cn/) or The Cancer Genome Atlas (TCGA) platforms. In detail, the analysis of alteration frequencies of *MLL3* in multiple cancers were carried out via cBioportal platform. The overall survival analysis (OS) and disease-free survival (DFS, also called relapse-free survival and RFS) were carried out via GEPIA platform (http://gepia.cancer-pku.cn/). The high/low cutoffs were 50%. The log-rank test was used for hypothesis evaluation. Values of *p* < 0.05 were considered as significant differences. The gene expression correlation analysis was performed with GEPIA using TCGA datasets.

### Statistical analysis

For all the experimental studies, the assays were repeated at least with three biological replicates. Data were shown as average values ± SD or SEM. *P*-value was calculated using Student’s *t* test, and statistical significance was assigned with *P-value < 0.05, **P-value < 0.01, ***P-value < 0.001.

## Results

### MLL3 is frequently mutated in multiple cancers

**Fig. 1:**
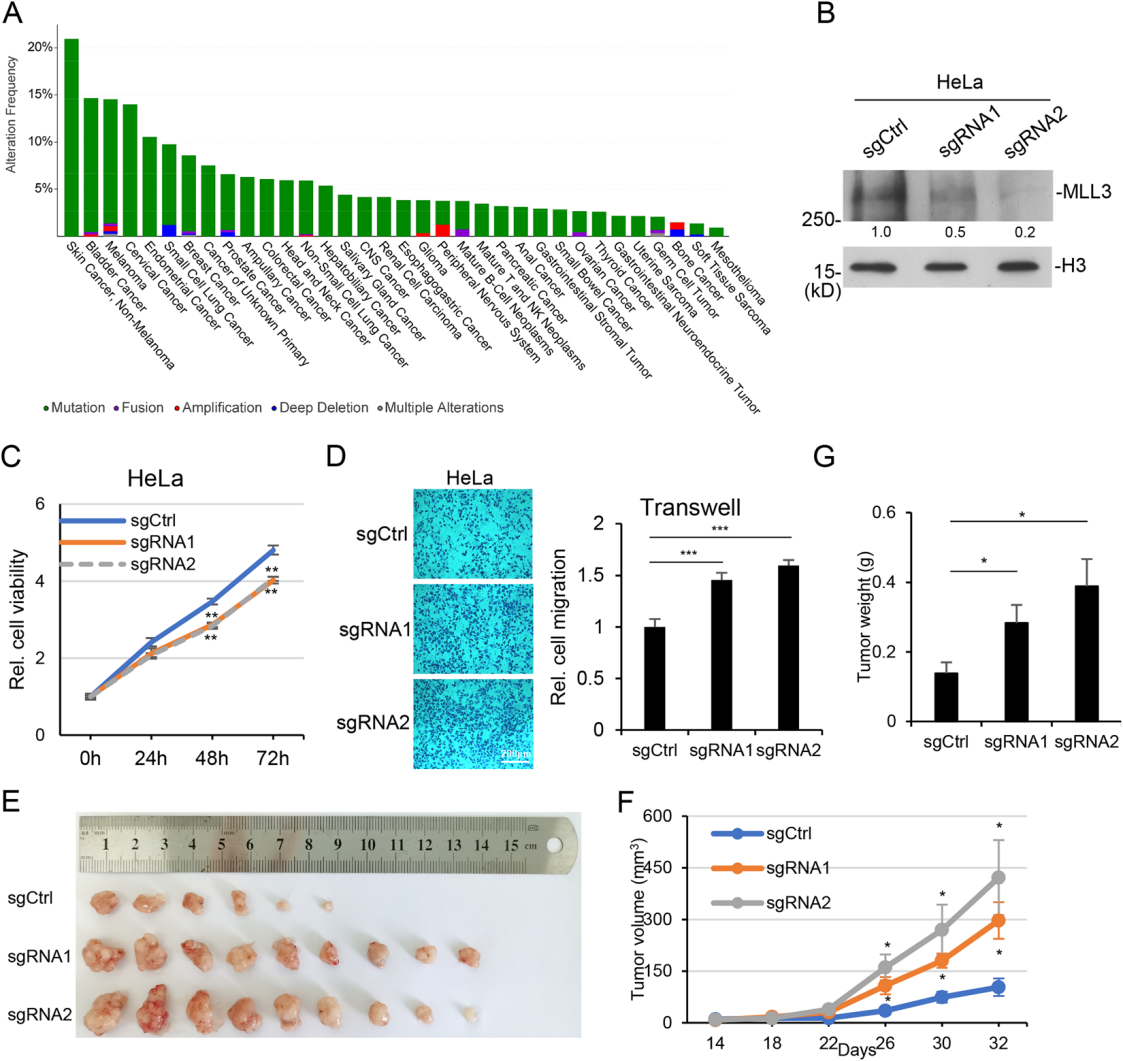
MLL3 regulates cancer cell migration. **A** Histogram showing the mutation rates of MLL3 in different types of cancer. Data are from MSK-IMPACT Clinical Sequencing Cohort (MSKCC, Nat Med 2017). **B** Two independent *MLL3* knockdown HeLa cell lines were generated with CRISPR/Cas9 system. MLL3 protein levels were examined by western blotting. **C** Cells were prepared as (**B**). Cell viability assay was performed to examine the cell proliferation rates after MLL3 depletion. **D** Cells were prepared as (**B**) and transwell assay was performed to measure cell migration after MLL3 depletion. **E**–**G** Cells were prepared as (**B**) and injected into nude mice. Tumors were pictured (**E**). Tumor growth curves (**F**) and tumor weight (**G**) were shown as mean ± SEM (*n* = 6,9,9 respectively). *P < 0.05, **P < 0.01, ***P < 0.001.

MLL3 is one of the key enzymes for enhancer activity, transcription regulation, and multiple developmental and pathological processes. We searched TCGA database and found that *MLL3* is frequently mutated in many types of cancers, including skin cancers, bladder cancer, cervical cancer, and so on (Fig. 1A). It is also lower expressed in some types of cancers, such as cervical squamous cell carcinoma and endocervical adenocarcinoma (CESC), uterine corpus endometrial carcinoma (UCEC), and colon adenocarcinoma (COAD) (Sup. Fig. S1A). Its lower expression is associated with poor prognosis in kidney renal clear cell carcinoma (KIRC) (Sup. Fig. S1B).

### MLL3’s functions in cancer cell growth and migration

To verify MLL3’s function in cancer, we constructed MLL3 knockout cell lines in HeLa cells with CRISPR/sgRNA system. Two independent sgRNAs were used in the following experiments (Fig. 1B). The results of cell viability and transwell assays indicated that MLL3 depletion repressed cell proliferation rate slightly, and improved cell migration (Fig. 1C, D). The experiments were performed in four different cancer cell lines, which showed the same results, including U2OS, an osteosarcoma cell line, HeLa, a cervical cancer cell line, 769-P, a kidney cancer cell line, and MDA-MB-231, a breast cancer cell line (Sup. Fig. S2). The results seemed contradictory, and to further clarify it, we performed xenograft experiments and injected the above cells into nude mice. Since the rate of tumor formation in xenograft experiments for U2OS is quite low, we used HeLa here. The result showed that MLL3-depleted cells grew faster in mice and formed bigger tumors (Fig. 1E–G). These strongly supported *MLL3* as a tumor suppressor, which is consistent with TCGA data. It seemed that MLL3 deficiency did not enhance cell proliferation in our system, however, we cannot exclude the possibility that MLL3 deficiency may cause a dramatic effect on cell growth in other cell lines or under certain treatments.

### MLL3 selectively regulates the expression of p53 target genes

It was reported previously MLL3 is involved in p53 signaling^36^. U2OS is a cell line with wild-type p53 and widely used in p53-related studies, so we chose U2OS cells to investigate the underlying mechanisms. we activated p53 with Nutlin-3A treatment in U2OS cells and examined the effects of *MLL3* deficiency. *MLL3* knockdown with siRNAs successfully attenuated the activation of *MDM2* and *p21/CDKN1A*, but not *PUMA/BBC3* (Sup. Fig. S3A, B). RNA-Seq was performed to investigate MLL3’s effect on gene expression. A heatmap was generated to show that *MLL3* deficiency only impairs the activation of a subgroup of p53 target genes, including *MDM2* and *p21/CDKN1A* (Sup. Fig. S3C, upper group, Sup. Table 1). The results of ChIP assay indicated that enrichment of p53 on *p21* promoter and enhancer was reduced in *MLL3* knockdown cells (Sup. Fig. S3D–G). These are consistent with the previous studies that MLL3 is involved in the activation of p53 target genes.

### MLL3 deficiency enhances gene expression associated with migration

**Fig. 2:**
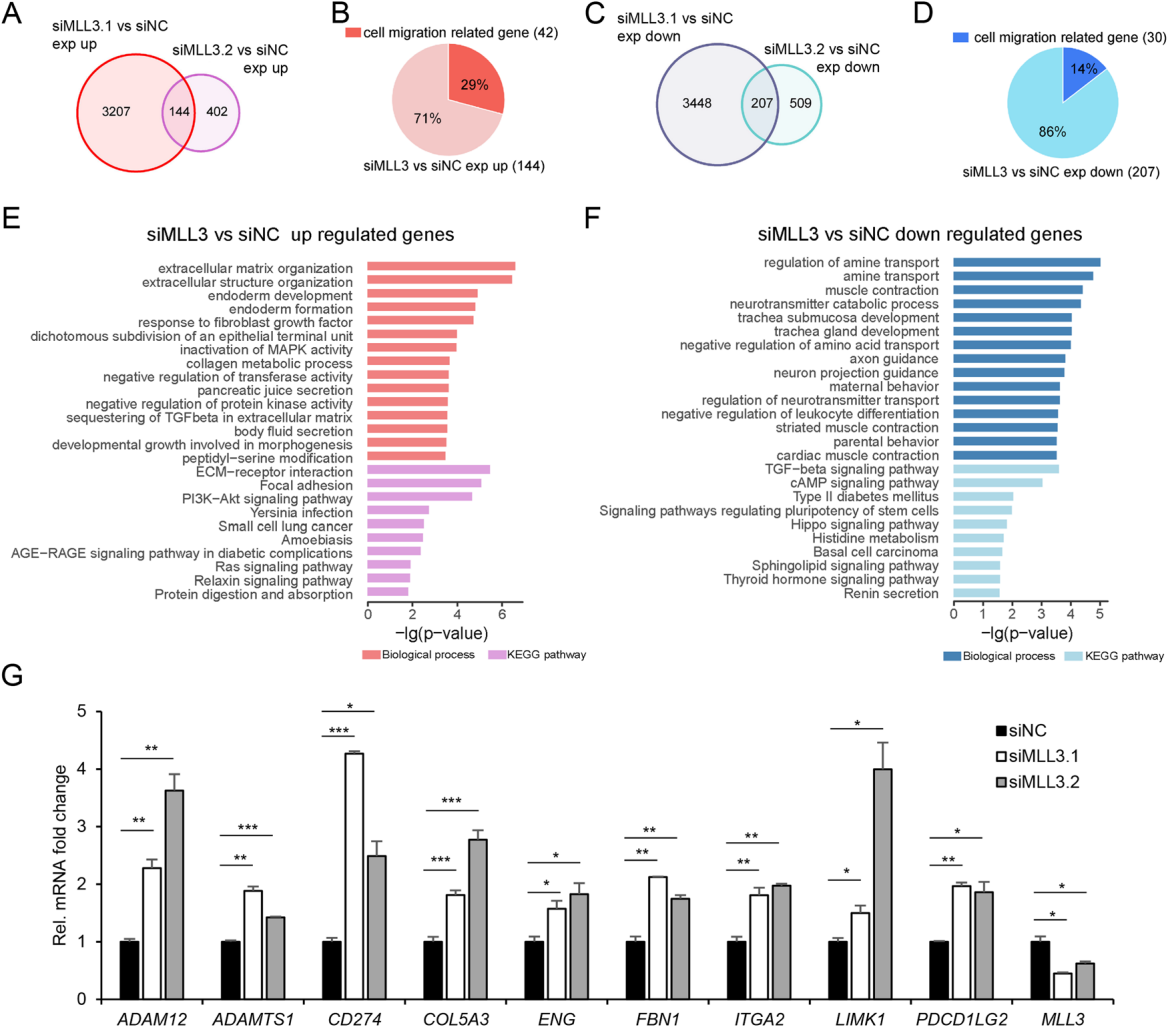
Gene expression profiles regulated by MLL3. **A** *MLL3* was knocked down with two different siRNAs in U2OS cells. DEGs (fold change > 1.5) between control and each siRNA were identified. Venn Diagrams show the number of upregulated genes between control and two *MLL3* siRNAs. **B** The percentage of cell migration-related genes in the overlapped upregulated genes. **C** Venn Diagrams show the number of downregulated DEGs (fold change > 1.5) between control and two *MLL3* siRNAs. **D** The percentage of cell migration-related genes in downregulated genes. **E**, **F** Biological process and KEGG pathway enrichment analyses of upregulated DEGs (**E**) or downregulated DEGs (**F**) were performed with DAVID, and items were ordered by *p* value. **G** Verification of chosen upregulated cell-migration-related DEGs after *MLL3* knockdown by qRT-PCR. Histograms show the relative mRNA fold change presented as mean ± SD (*n* = 3). *P < 0.05, **P < 0.01, ***P < 0.001.

The above data indicated that MLL3 acts as a tumor suppressor mainly through regulating migration but not cell proliferation. Since p53 signaling is mainly involved in cell cycle arrest and apoptosis, we considered MLL3 maybe regulates cell migration through other mechanisms. To explore it, we analyzed RNA-Seq analysis in U2OS cells with MLL3 knockdown by two different siRNAs and identified the different expressed genes (DEGs) after a knockdown by each siRNA. Then we overlapped the DEGs and considered the overlapped genes were potential MLL3 target genes. Totally we identified 144 upregulated genes and 207 downregulated genes (Fig. 2A–D, Sup. Table 2). The functional annotation indicated that the upregulated DEGs are mainly associated with extracellular matrix organization and focal adhesion, which are known to be involved in cell migration (Fig. 2E, F). We verified the expression of some genes with quantitative RT-PCR (Fig. 2G). These results were consistent with our previous result about MLL3’s function in migration (Fig. 1D).

### Identification of MLL3 target genes through high-throughput sequencing

**Fig. 3:**
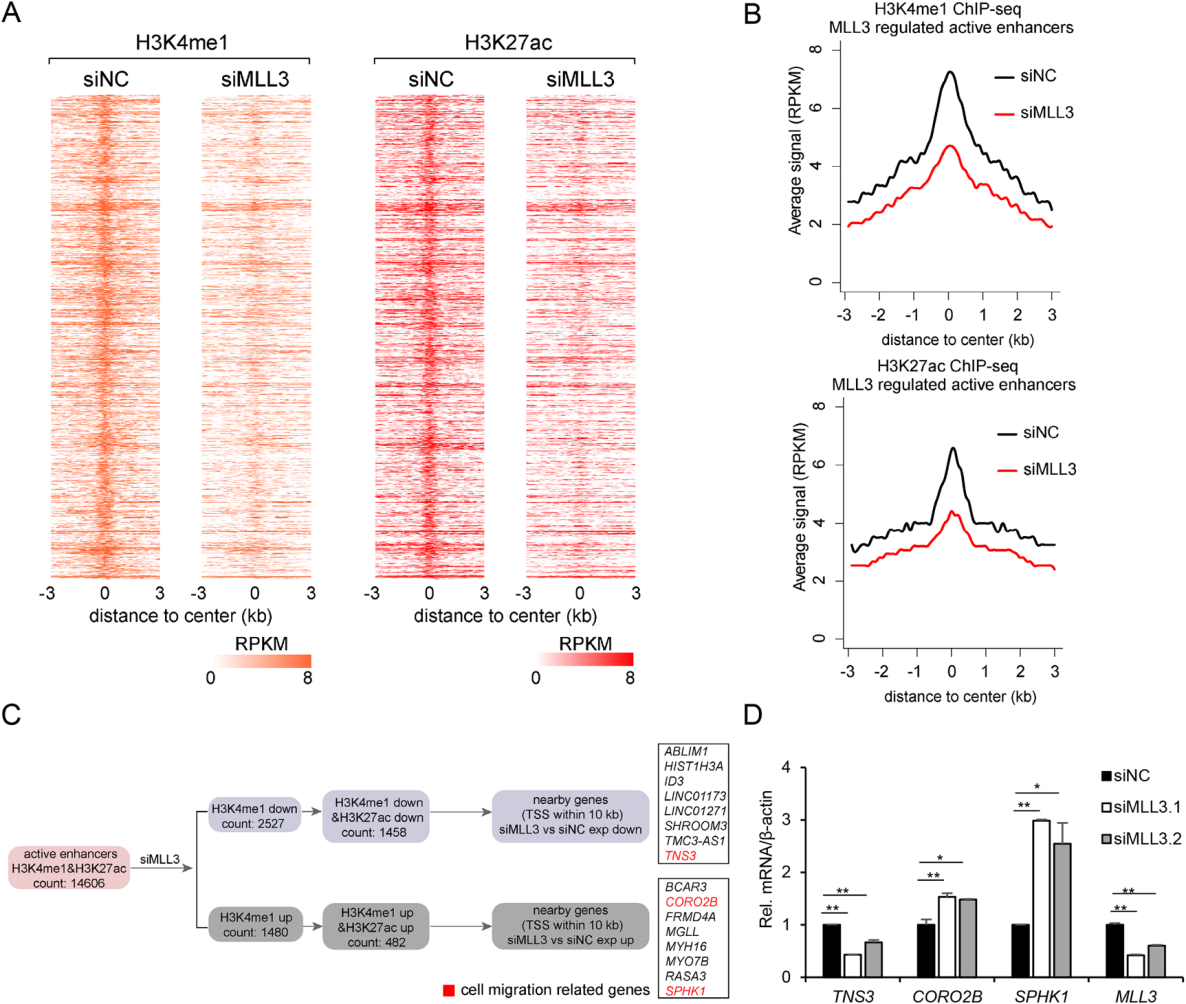
ChIP-Seq analysis to identify MLL3 target genes. **A** Heatmaps showing H3K4me1 and H3K27ac signals (RPKM) over 1458 MLL3 regulated active enhancer regions in siNC and siMLL3 cells. Heatmaps are sorted by the strength of H3K4me1 signals. **B** Aggregate plots comparing the average signals (RPKM) of H3K4me1 and H3K27ac on the enhancer regions in (**A**). **C** A sketch map showing the pipeline to identify enhancers regulated by MLL3 and their nearby genes. Genes related to cell migration are highlighted in red. **D** Verification of the expression of red highlighted genes in (**C**) after *MLL3* knockdown by qRT-PCR. Histograms show the relative mRNA fold change presented as mean ± SD (*n* = 3). *P < 0.05, **P < 0.01, ***P < 0.001.

To identify the target genes of MLL3, the best approach is to perform ChIP-Seq analysis with MLL3 antibody. But the antibodies in our hand were not good enough for ChIP analysis. So, we performed ChIP-Seq of H3K4me1 and H3K27ac. Since MLL3 plays critical role in enhancer regulation, its direct target loci should have lower signal of H3K4me1 and H3K27ac after MLL3 knockdown. Our ChIP-Seq analysis totally identified 14606 active enhancer loci in U2OS cells; after MLL3 knockdown, 2527 enhancers showed lower H3K4me1 and 1458 enhancers had lower signals of the above two modifications (Fig. 3A–C). Heatmaps were presented to show the levels of these modifications on the above 1458 enhancers (Fig. 3A), and their average levels on the enhancers were shown in Fig. 3B. To identify MLL3 target genes, we overlapped downregulated DEGs with the repressed enhancer loci, and identified 8 genes, among which *TNS3* is a gene reported to repress migration (Fig. 3C). The regulation of *TNS3* expression by MLL3 was further confirmed by quantitative RT-PCR (Fig. 3D). Considering MLL3’s role in transcription and cancer, we deduced that *TNS3* probably is the direct target gene for MLL3 to regulate cell migration.

### MLL3 regulates TNS3 expression in multiple cancer cell lines

**Fig. 4:**
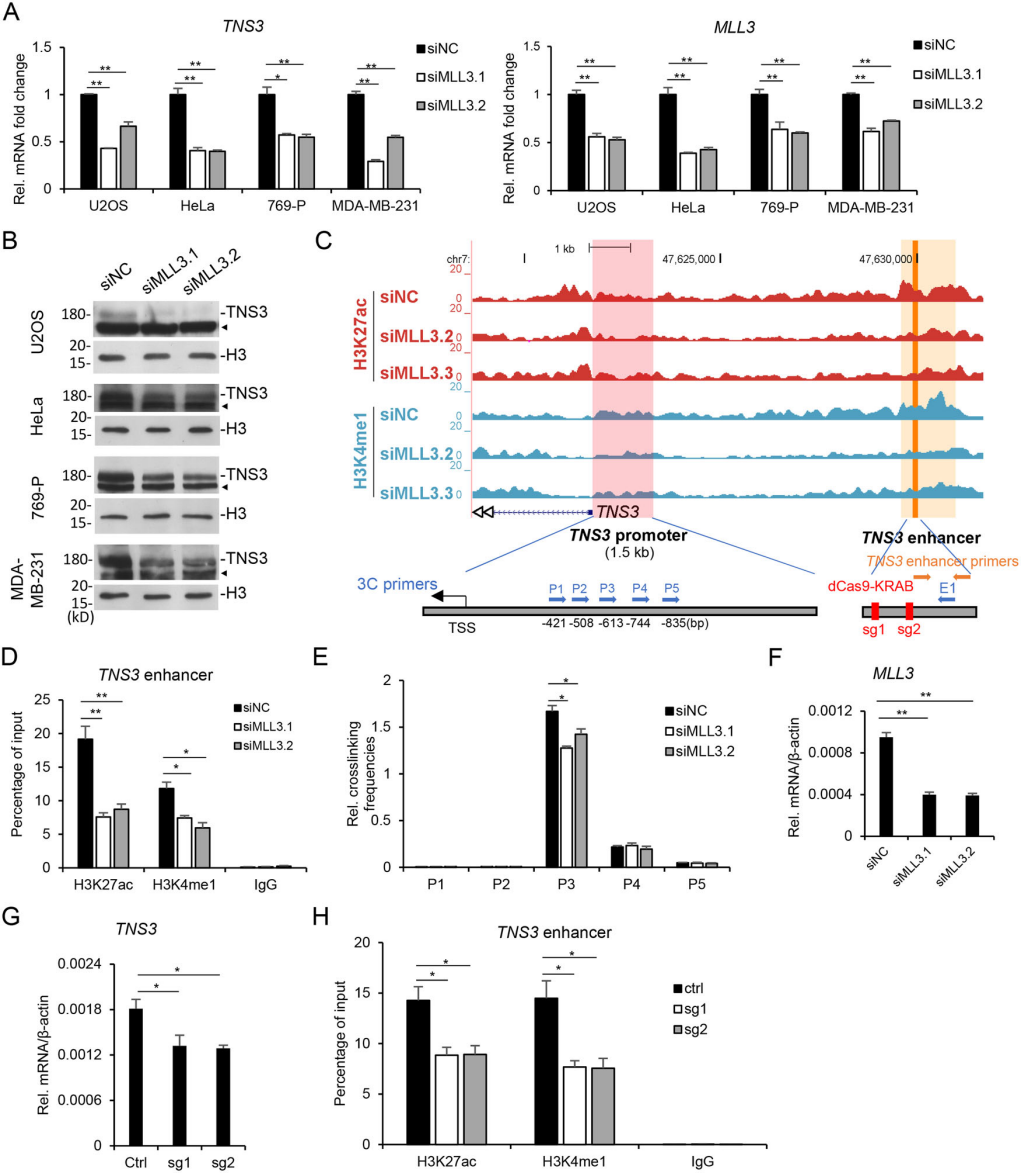
MLL3 targets *TNS3* enhancer. **A** *MLL3* was knocked down with two different siRNAs in U2OS, HeLa, 769-P, and MDA-MB-231 cell lines. The mRNA levels of *TNS3* and *MLL3* were determined by qRT-PCR. **B** Cells were prepared as (**A**). TNS3 proteins were analyzed by western blotting. **C** The genome browser view showing H3K27ac and H3K4me1 enrichment on *TNS3* promoter and enhancer regions after *MLL3* knockdown. The *TNS3* promoter and enhancer regions are highlighted in pink and orange respectively. The orange arrows indicate the loci of *TNS3* enhancer primers. The blue arrows indicate the loci of 3C primers. The red bars indicate the loci targeted by dCas9-KRAB sgRNAs. **D** U2OS cells were prepared as (**A**) and ChIP qRT-PCR analysis was performed to determine the relative enrichment of H3K27ac and H3K4me1 (percentage of input) on *TNS3* enhancer. **E** U2OS cells were prepared as (**A**) and 3C assay was performed. The relative crosslinking frequencies were determined by qRT-PCR. **F** *MLL3* mRNA levels in the cells used in (**D**) and (**E**) were determined by qRT-PCR. **G**, **H** *TNS3* enhancer activity was repressed by dCas9-KRAB/sgRNA system. *TNS3* mRNA levels were determined by qRT-PCR (**G**) and ChIP assay was performed to measure the modifications on the enhancer (**H**). All the data are presented as mean ± SD (*n* = 3). *P < 0.05, **P < 0.01. ◄ represents unspecific bands.

Since MLL3 acts as a tumor-suppressive gene in multiple cancer types, we examined its function on *TNS3* in several cancer cell lines, including U2OS, HeLa, 769-P, and MDA-MB-231 (Fig. 4A). In all the above cell lines, *MLL3* knockdown repressed *TNS3* expression (Fig. 4A), the downregulation of TNS protein level was also confirmed by western blotting (Fig. 4B). These indicate that the regulation of MLL3 on TNS3 expression is quite common in cancer cells, which is consistent with the previous study (Sup. Fig. S2).

### MLL3 regulates the activity of TNS3 enhancer

After surveying the ChIP-Seq results, we found an enhancer locus around 7 kb ahead of *TNS3* TSS, with lower H3K4me1 and H3K27ac signal in *MLL3* knockdown cells (Fig. 4C). To prove it as the MLL3 target enhancer for *TNS3*, we first confirmed the reduction of two modifications on it after *MLL3* knockdown with ChIP-PCR (Fig. 4D). Then 3C assay was performed and verified the interaction between the predicted enhancer and *TNS3* promoter. We designed one primer in the enhancer and five primers in the promoter region (Fig. 4C, blue color), and detected a positive signal using P3 and enhancer primer pair (Fig. 4E, F). The result indicated that although the distance between the sequences of P3 and E1 is around 7 kb on chromatin, their spatial distance is very short in cells and the two DNA fragments interact with each other. *MLL3* knockdown also reduced the 3C signal, indicating MLL3 is involved in the chromatin interaction (Fig. 4E). To further investigate the function of TNS3 enhancer, we utilized a dCas9-KRAB/sgRNA system which could be used to repress specific enhancer activity. Transfection of two different sgRNAs both significantly inhibited H3K27ac and H3K4me1 on the enhancer, and repressed *TNS3* expression (Fig. 4G, H). These data together indicated that MLL3 regulates the histone modifications, activity, and chromatin interaction of *TNS3* enhancer, and in turn controls *TNS3* expression.

### MLL3 regulates cell migration through TNS3

**Fig. 5:**
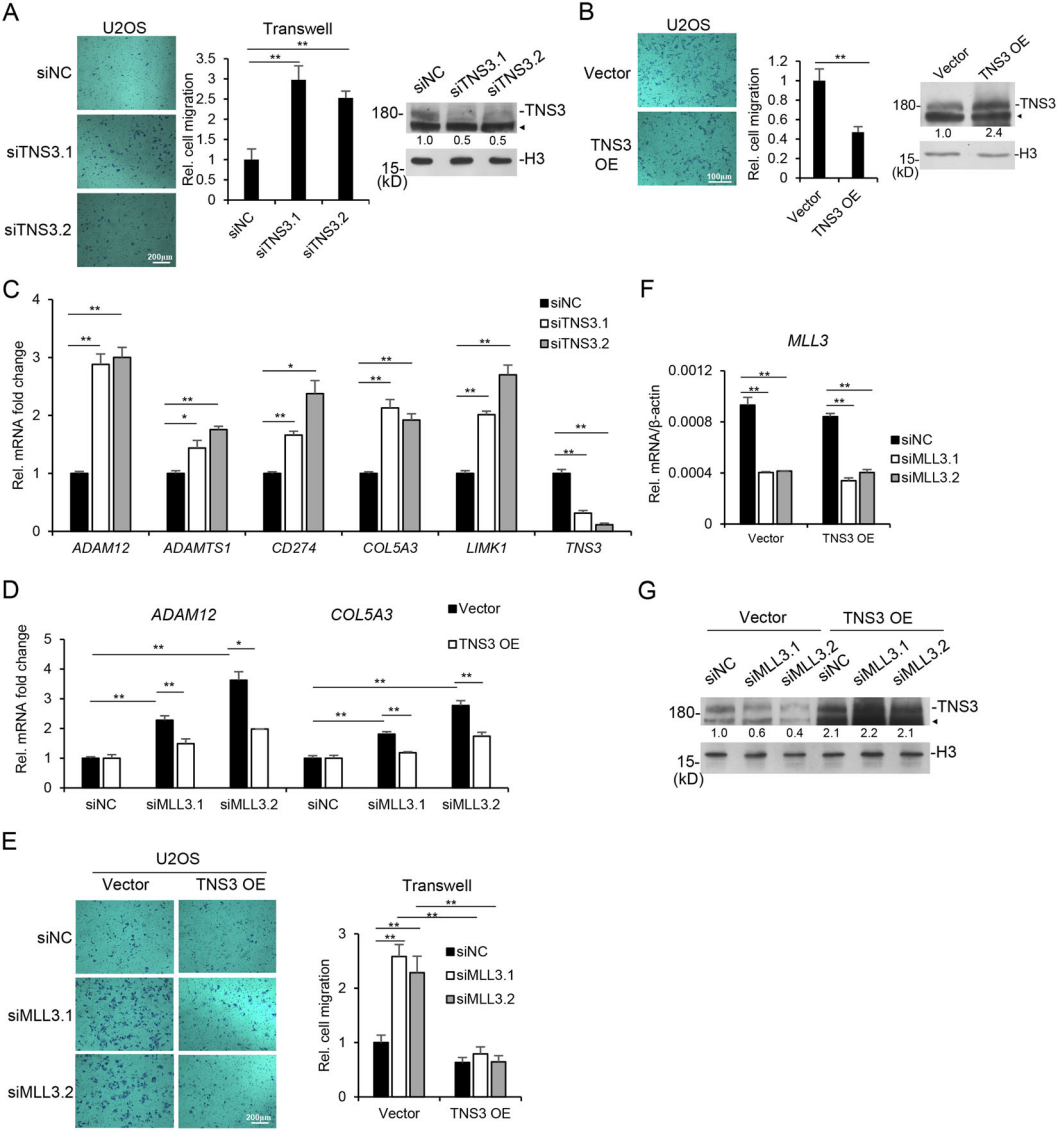
MLL3 deficiency increased cell migration through TNS3. **A** *TNS3* was knocked down with siRNAs in U2OS cells. Transwell assay was performed to show cell migration with or without TNS3. **B** TNS3 was exogenous expressed in U2OS cells, and transwell assay was performed to measure cell migration. TNS3 proteins were analyzed by western blotting with anti-TNS3 antibody. **C** *TNS3* was knocked down with two different siRNAs, and the mRNA levels of *ADAM12*, *ADAMTS1*, *CD274*, *COL5A3*, *LIMK1*, and *TNS3* were determined by qRT-PCR. **D** *MLL3* was knocked down and then TNS3 was exogenous expressed in U2OS cells. The mRNA levels of *ADAM12* and *COL5A3* were determined by qRT-PCR. **E** Cells were treated as (**D**) and transwell assay was performed to measure cell migration. Histogram showing the statistical calculation of relative cell migration. **F**, **G** *MLL3* mRNA levels and TNS3 protein of the cells used in (**D**, **E**) were determined by qRT-PCR (**F**) and western blotting (**G**), respectively. All the data are presented as mean ± SD (*n* = 3). *P < 0.05, **P < 0.01. ◄ represents unspecific bands.

Previously we showed that MLL3 deficiency increased cell migration in HeLa cells (Fig. 1D). *TNS3* was reported as a gene involved in migration and has been associated with several types of cancers^37–39^. As expected, *TNS3* knockdown increased cell migration (Fig. 5A), and its over-expression repressed it (Fig. 5B). Furthermore, *TNS3* depletion increased the expression of several migration-related genes, which were identified in *MLL3* knockdown RNA-Seq, such as *ADAM12*, *ADAMTS1*, *CD274*, *COL5A3*, *LIMK1* (Figs. 2G and 5C). Consistently, the enhanced expression of *ADAM12* and *COL5A3* by *MLL3* knockdown was repressed when *TNS3* was exogenous expressed (Fig. 5D). In the above cells, exogenous expression of *TNS3* significantly inhibited cell migration and invasion ability enhanced by *MLL3* knockdown (Fig. 5E–G, Sup. Fig. 4). These indicated that MLL3 controls cell migration and migration-related gene expression through TNS3.

### TNS3 is lower expressed in multiple types of cancers

**Fig. 6:**
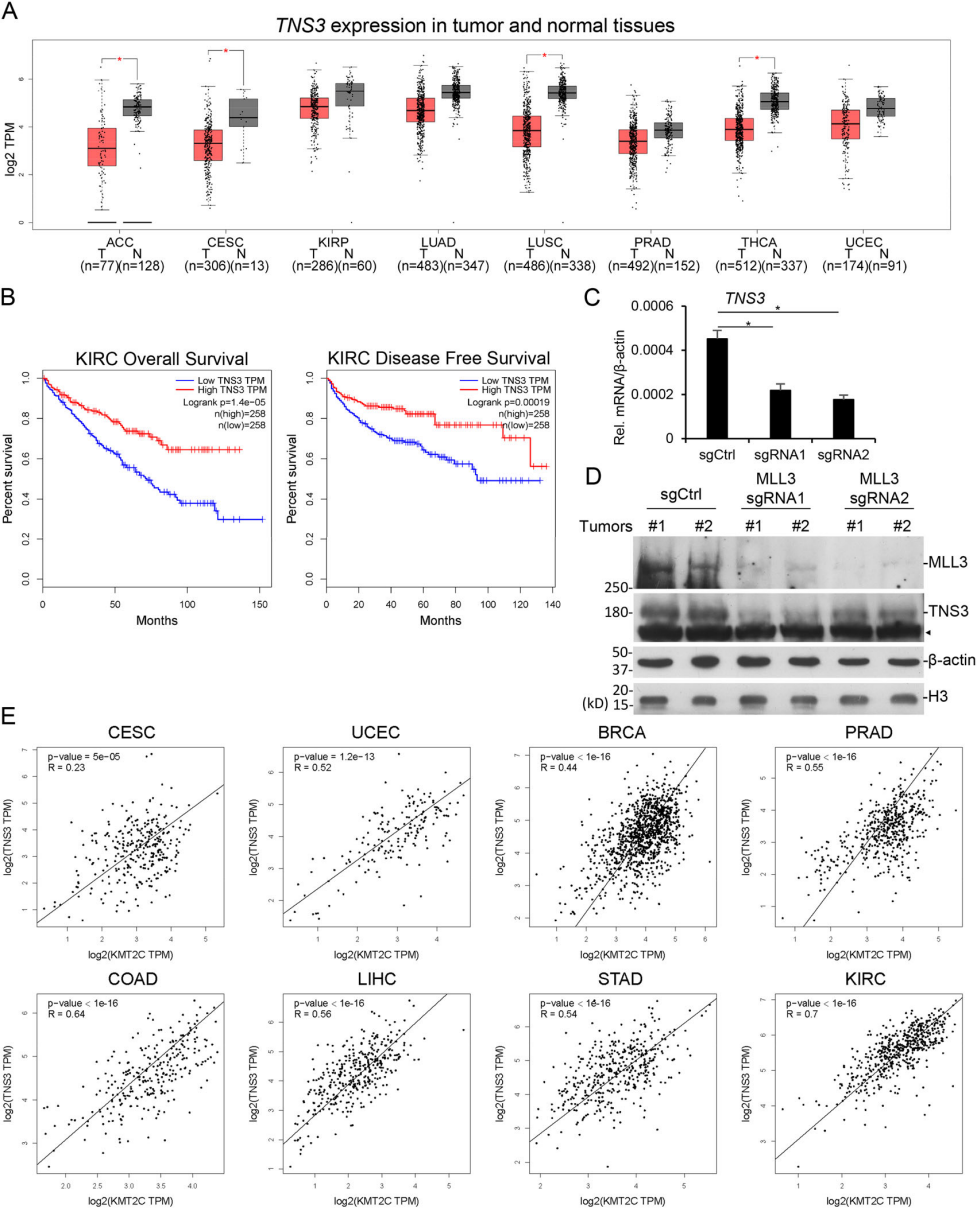
Correlation of *MLL3* and *TNS3* in cancer patient tissues. **A** Boxplots showing the expression (log2 TPM) of TNS3 in multiple tumor and normal tissues in TCGA database. **B** The TCGA RNA-seq data of kidney cancer tissues were analyzed. Overall survival (OS) and disease-free survival (DFS) were analyzed and plotted using the Kaplan–Meier method. The survival rates for patients with high or low TNS3 expression are plotted as red or blue lines, respectively. The number of patients in each group is shown in parentheses. p-Values were calculated using a log-rank test. **C** The mRNA levels of TNS3 of tumors shown in (Fig. 1E) were determined by qRT-PCR. Three tumors from each group were randomly picked. Histograms are presented as mean ± SEM (*n* = 3). **D** Two tumors from each group were random picked and the protein levels of MLL3 and TNS3 were analyzed by WB with indicated antibodies. **E** The correlation of *MLL3/KMT2C* and *TNS3* in multiple cancer tissues from TCGA datasets was analyzed. *p*-Values were calculated using a log-rank test. *P < 0.05. ◄ represents unspecific bands.

To investigate the relationship between *TNS3* and cancer, we investigated TCGA database and found that *TNS3* is lower expressed in many types of cancer, including adrenocortical carcinoma (ACC), CESC, kidney renal papillary cell carcinoma (KIRP), lung adenocarcinoma (LUAD), lung squamous cell carcinoma (LUSC), prostate adenocarcinoma (PRAD), thyroid carcinoma (THCA), and UCEC (Fig. 6A). Like *MLL3*, low expression of *TNS3* is also associated with poor prognosis in KIRC (Fig. 6B). These suggested *TNS3* acts as a tumor suppressor in multiple cancers.

### MLL3 expression is highly correlated with TNS3 in multiple cancers

To further investigate the relationship between MLL3 and TNS3 in cancers, we examined the protein expression in the tumors derived from *MLL3* deficient HeLa cells (Fig. 1E). In these tumors, *TNS3* mRNA and protein levels were greatly reduced when *MLL3* was depleted (Fig. 6C, D). Investigation of TCGA datasets also indicated that *MLL3* and *TNS3* are positively correlated in multiple types of cancers, including CESC, UCEC, breast invasive carcinoma (BRCA), PRAD, COAD, liver hepatocellular carcinoma (LIHC), stomach adenocarcinoma (STAD) and KIRC. These data together indicate that *TNS3* is the target gene for MLL3 to suppress cancer.

## Discussion

MLL3 is one of the key enzymes for the modifications and activity of enhancers. It is frequently mutated in many cancers. Previously, it was reported to be associated with p53 and regulates the expression of p53 target genes. Our results indicated that besides p53 signaling, MLL3 also regulates tumorigenesis through repressing cell migration. MLL3 regulates H3K4me1 and H3K27ac on *TNS3* enhancer, which in turn affects *TNS3* transcription. *TNS3* has been shown as an important protein involved in integrin-mediated signaling and cell migration^37^. The gene expression and DNA methylation on CpG island of *TNS3* have been associated with breast cancer and renal cell carcinoma^38,39^. We showed that *TNS3* exogenous expression completely blocked the elevated cell migration caused by *MLL3* knockdown, indicating the critical role of TNS3 in MLL3’s function in repressing cell migration. Though *MLL3* or *TNS3* knockdown did not elevate downstream gene dramatically, quite a few migration-related genes were upregulated. A mechanism published previously involving JNK/cJun pathway is probably also functioning here^37^.

Enhancer regulation is one of the important steps in transcription regulation. One gene is controlled by multiple enhancers and one enhancer can target multiple genes. Their relationship varies dependent on the context of the cell environment. To clarify the dynamics of enhancer activity and target genes are helpful to identify novel targets for diagnosis and treatment. Our study identified the enhancer for *TNS3* gene in *MLL3* deficient cells, which will be important to understand how MLL3 suppresses cancer and the related future studies.

Our data support MLL3 as a regulator for p53 signaling, which is consistent with the previous report^36,40^. Moreover, we found that MLL3 selectively regulates p53 target genes, such as *MDM2* and *p21*, but not *PUMA* (Sup. Fig. 3). We also noticed that *MLL3* deficiency did not completely repress activation of p53 target genes, nor did it increase cell proliferation (Fig. 1C and Sup. Fig. 3). These suggest that at least in the tested cell lines, *MLL3* depletion promoted tumorigenesis mainly through cell migration but not proliferation. It will be interesting to further investigate the mechanisms for MLL3 regulating p53 signaling and its roles in cancer.

### Data access

The high-throughput sequencing data have been submitted to GEO database (Acc. No. GSE160254).

## Supplementary information

Supplemental Figures

Supplemental Tables

## References

[1] Lee NK, Fong PK, Abdullah MT (2014). Modelling complex features from histone modification signatures using genetic algorithm for the prediction of enhancer region. Bio-Med. Mater. Eng..

[2] Yuan J (2017). Super-enhancers promote transcriptional dysregulation in nasopharyngeal carcinoma. Cancer Res..

[3] Roe JS (2017). Enhancer reprogramming promotes pancreatic cancer metastasis. Cell.

[4] Ooi WF (2016). Epigenomic profiling of primary gastric adenocarcinoma reveals super-enhancer heterogeneity. Nat. Commun..

[5] Akhtar-Zaidi B (2012). Epigenomic enhancer profiling defines a signature of colon cancer. Science.

[6] Calo E, Wysocka J (2013). Modification of enhancer chromatin: what, how, and why?. Mol. Cell.

[7] Rickels R, Shilatifard A (2018). Enhancer logic and mechanics in development and disease. Trends Cell Biol..

[8] Medina-Rivera A, Santiago-Algarra D, Puthier D, Spicuglia S (2018). Widespread enhancer activity from core promoters. Trends Biochem. Sci..

[9] Murakawa Y (2016). Enhanced Identification of transcriptional enhancers provides mechanistic insights into diseases. Trends Genet..

[10] Creyghton MP (2010). Histone H3K27ac separates active from poised enhancers and predicts developmental state. Proc. Natl Acad. Sci. USA.

[11] Hnisz D (2013). Super-enhancers in the control of cell identity and disease. Cell.

[12] Mack SC (2018). Therapeutic targeting of ependymoma as informed by oncogenic enhancer profiling. Nature.

[13] Shlyueva D, Stampfel G, Stark A (2014). Transcriptional enhancers: from properties to genome-wide predictions. Nat. Rev. Genet..

[14] Li QL (2019). The hyper-activation of transcriptional enhancers in breast cancer. Clin. Epigenet..

[15] Shen H (2016). Suppression of enhancer overactivation by a RACK7-histone demethylase complex. Cell.

[16] Lawrence MS (2014). Discovery and saturation analysis of cancer genes across 21 tumour types. Nature.

[17] Flavahan WA, Gaskell E, Bernstein BE (2017). Epigenetic plasticity and the hallmarks of cancer. Science.

[18] Yao J, Chen J, Li LY, Wu M (2020). Epigenetic plasticity of enhancers in cancer. Transcription.

[19] Wang L (2018). Resetting the epigenetic balance of Polycomb and COMPASS function at enhancers for cancer therapy. Nat. Med..

[20] Yao J (2020). GLIS2 promotes colorectal cancer through repressing enhancer activation. Oncogenesis.

[21] Wang HY (2019). Histone demethylase KDM3A is required for enhancer activation of hippo target genes in colorectal cancer. Nucleic Acids Res..

[22] Loven J (2013). Selective inhibition of tumor oncogenes by disruption of super-enhancers. Cell.

[23] Pott S, Lieb JD (2015). What are super-enhancers?. Nat. Genet..

[24] Wu M (2008). Molecular regulation of H3K4 trimethylation by Wdr82, a component of human Set1/COMPASS. Mol. Cell Biol..

[25] Cho YW (2007). PTIP associates with MLL3- and MLL4-containing histone H3 lysine 4 methyltransferase complex. J. Biol. Chem..

[26] Sze CC, Shilatifard A (2016). MLL3/MLL4/COMPASS family on epigenetic regulation of enhancer function and cancer. Cold Spring Harb. Perspect. Med..

[27] Wang C (2016). Enhancer priming by H3K4 methyltransferase MLL4 controls cell fate transition. Proc. Natl Acad. Sci. USA.

[28] Herz HM (2012). Enhancer-associated H3K4 monomethylation by Trithorax-related, the *Drosophila* homolog of mammalian Mll3/Mll4. Genes Dev..

[29] Lai B (2017). MLL3/MLL4 are required for CBP/p300 binding on enhancers and super-enhancer formation in brown adipogenesis. Nucleic Acids Res..

[30] Hu D (2013). The MLL3/MLL4 branches of the COMPASS family function as major histone H3K4 monomethylases at enhancers. Mol. Cell Biol..

[31] Lee JE (2013). H3K4 mono- and di-methyltransferase MLL4 is required for enhancer activation during cell differentiation. eLife.

[32] Yan J (2018). Histone H3 lysine 4 monomethylation modulates long-range chromatin interactions at enhancers. Cell Res..

[33] Dorighi KM (2017). Mll3 and Mll4 facilitate enhancer RNA synthesis and transcription from promoters independently of H3K4 monomethylation. Mol. Cell.

[34] Zhu K (2017). SPOP-containing complex regulates SETD2 stability and H3K36me3-coupled alternative splicing. Nucleic Acids Res..

[35] Zhao QY (2016). Global histone modification profiling reveals the epigenomic dynamics during malignant transformation in a four-stage breast cancer model. Clin. Epigenet..

[36] Lee J (2009). A tumor suppressive coactivator complex of p53 containing ASC-2 and histone H3-lysine-4 methyltransferase MLL3 or its paralogue MLL4. Proc. Natl Acad. Sci. USA.

[37] Park GC (2019). Tensin-3 regulates integrin-mediated proliferation and differentiation of tonsil-derived mesenchymal stem cells. Cells.

[38] Vess A (2017). A dual phenotype of MDA-MB-468 cancer cells reveals mutual regulation of tensin3 and adhesion plasticity. J. Cell Sci..

[39] Carter JA, Gorecki DC, Mein CA, Ljungberg B, Hafizi S (2013). CpG dinucleotide-specific hypermethylation of the TNS3 gene promoter in human renal cell carcinoma. Epigenetics.

[40] Kim DH, Kim J, Lee JW (2011). Requirement for MLL3 in p53 regulation of hepatic expression of small heterodimer partner and bile acid homeostasis. Mol. Endocrinol..

