# Supplementary material for: MLL3 suppresses tumorigenesis through regulating TNS3 enhancer activity
Source: Cell Death Dis. 2021 Apr 6;12(4):364. doi: 10.1038/s41419-021-03647-2 (PMC8024252; doi:10.1038/s41419-021-03647-2)
Supplement: Supplementary file 1 — Supplemental Figures [file 41419_2021_3647_MOESM1_ESM.pdf]

Sup. Figure S1

A

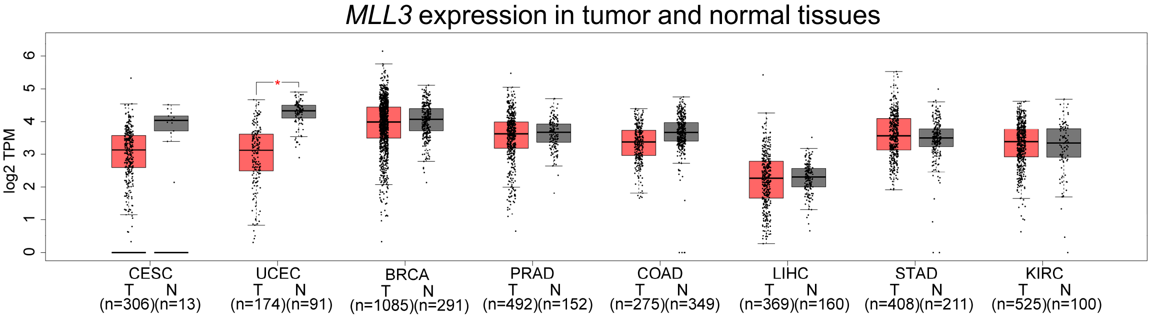

B

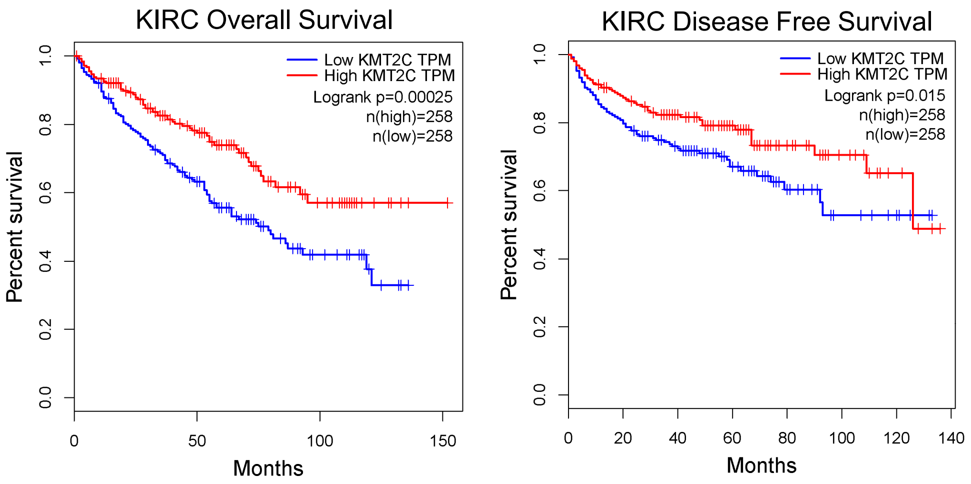

Sup Fig. S1 MLL3 expression in cancer tissues. (A) Boxplots showing the TCGA expression (log2 TPM) of *MLL3* in multiple tumor and normal tissues. **(B)** The TCGA RNA-seq data of kidney cancer tissues were analyzed. Overall survival (OS) and disease-free survival (DFS) were analyzed and plotted using the Kaplan–Meier method. The survival rates for patients with high or low *MLL3* expression are plotted as red or blue lines, respectively. The number of patients in each group is shown in parentheses. P-Values were calculated using a log-rank test.

Sup. Figure S2

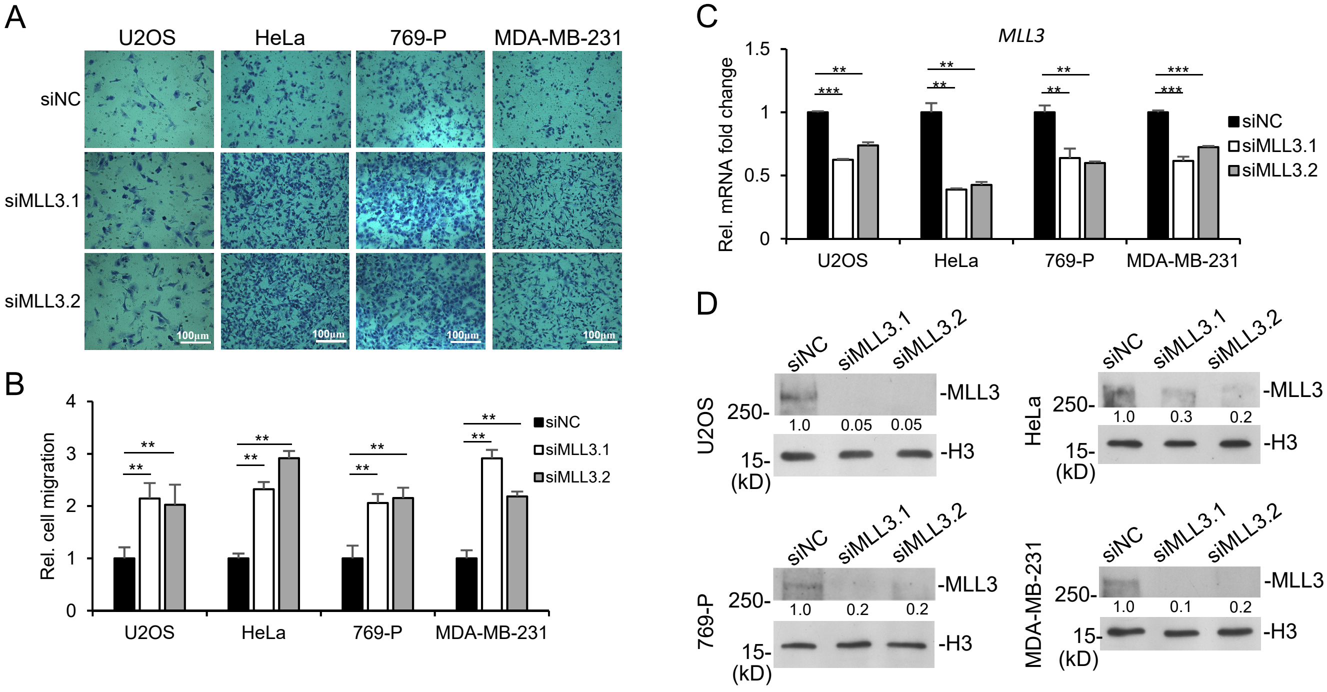

Sup. Figure S2 MLL3 deficiency increased migration of multiple cancer cell lines. (A) *MLL3* was knocked down by siRNAs in the indicated cells. Transwell assay was performed to measure cell migration after *MLL3* knockdown in four cell lines. **(B)** Histogram showing the statistical calculation of relative cell migration in (A). **(C)** The mRNA levels of *MLL3* were determined by qRT-PCR to confirm *MLL3* knockdown. **(D)** The protein levels of MLL3 were analyzed by western blotting with indicated antibodies. All the histograms are presented as mean ± SD (n=3).*P < 0.05, **P < 0.01, ***P < 0.001.

Sup. Figure S3

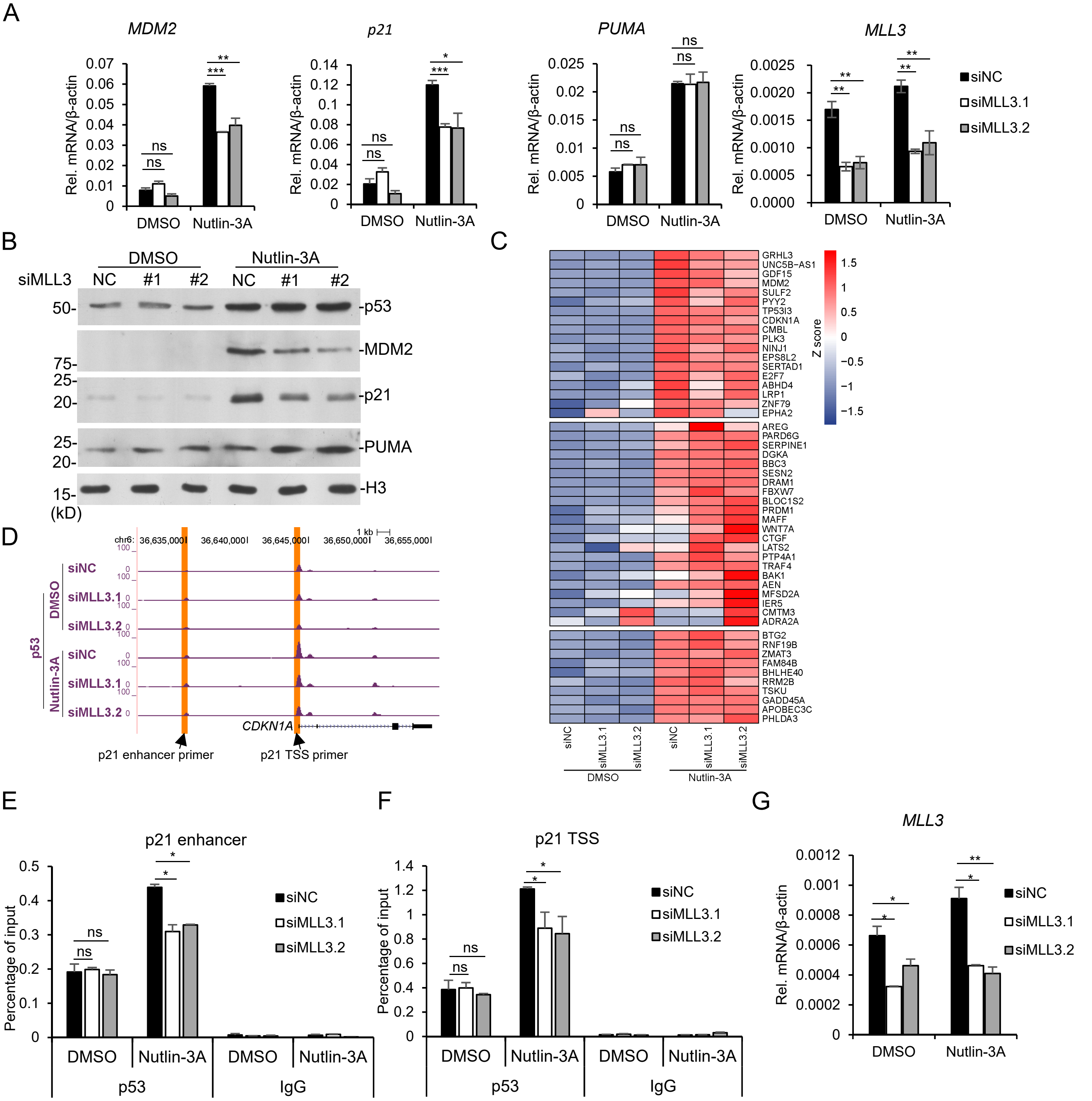

Sup Fig. S3 Regulation of p53 target genes by MLL3. (A) U2OS cells were transfected with control or two MLL3 siRNAs for 48h followed by treatment of DMSO or 20nm Nutlin-3A for 12h. The mRNA levels of *MDM2*, *p21*, *PUMA* and *MLL3* were determined by qRT-PCR. **(B)** Cells were prepared as (A). The protein levels of p53, MDM2, p21 and PUMA were analyzed by western blotting with indicated antibodies. **(C)** Heatmap showing the expression of p53 target genes after p53 activation and *MLL*3 knockdown. **(D)** The genome browser view showing p53 enrichment around *p21* after *MLL3* knockdown. The loci of *p21* TSS and enhancer primers are highlighted by orange bars. **(E&F)** Cells were prepared as (A) and ChIP assay was performed with anti-p53 antibody. The relative p53 enrichment (percentage of input) on *p21* TSS and enhancer were determined by qRT-PCR. **(G)** The mRNA levels of *MLL3* of cells used for ChIP in (E&F) were determined by qRT-PCR. All the histograms are presented as mean ± SD (n=3). *P < 0.05, **P < 0.01, ***P < 0.001.

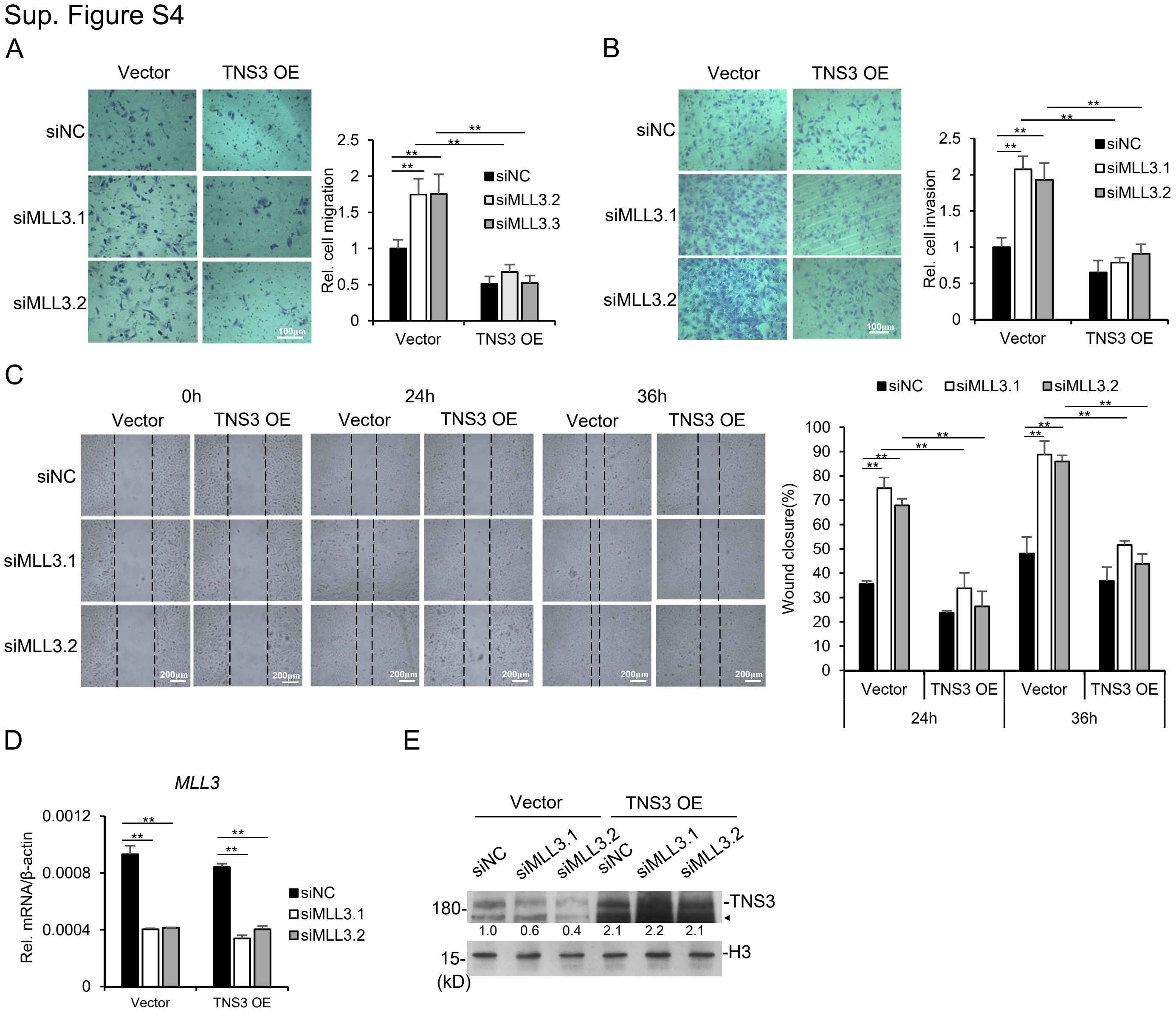

Sup. Fig. S4 Effects of MLL3 and TNS3 on migration and invasion. (A-C) U2OS cells were transfected with MLL3 siRNAs and TNS3 expressing plasmid, then examined with cell migration (A), invasion (B) and wound healing assays (C)**. (D&E)** MLL3 mRNA level and TNS3 protein level of cells were examined with real-time RT-PCR and western, respectively.
